# Anti-N-methyl-D-aspartate Receptor Encephalitis Associated with Ictal Torsades de Pointes and Cardiac Arrest

**DOI:** 10.7759/cureus.4837

**Published:** 2019-06-05

**Authors:** Faisal Inayat, Wikien A Hung Pinto, Soban Ahmad, Amna Hussain, Waqas Ullah

**Affiliations:** 1 Internal Medicine, Allama Iqbal Medical College, Lahore, PAK; 2 Internal Medicine, Einstein Medical Center, Philadelphia, USA; 3 Emergency Medicine, Hamad Medical Corporation, Doha, QAT; 4 Internal Medicine, Abington Hospital-Jefferson Health, Abington, USA

**Keywords:** anti-nmda receptor encephalitis, ictal torsades de pointes, cardiac arrest

## Abstract

Anti-N-methyl-D-aspartate (anti-NMDA) receptor encephalitis is a rare clinical entity that typically presents with psychiatric disturbances and neurological deficits. It is commonly associated with ovarian teratomas. Although these patients demonstrate a predilection to develop cardiac arrhythmias, such complications are frequently self-limited. We chronicle here a unique case of a young woman with adnexal teratoma who experienced a tonic-clonic seizure and cardiac arrest. Electrocardiogram showed polymorphic ventricular tachycardia, consistent with torsade de pointes. Based on extensive diagnostic workup and exclusion of probable etiologies, she was diagnosed with anti-NMDA receptor encephalitis. To the best of our knowledge, this report represents the first case of anti-NMDA receptor encephalitis complicated by ictal torsades de pointes, leading to cardiac arrest. This paper illustrates that patients with anti-NMDA receptor encephalitis can develop life-threatening cardiac dysrhythmia and cardiac arrest, requiring urgent management. Clinicians should be vigilant for severe autonomic dysfunction as prompt etiology establishment is of paramount importance in these patients.

## Introduction

In 2007, Dalmau et al. first described anti-N-methyl-D-aspartate (anti-NMDA) receptor encephalitis as a paraneoplastic manifestation of ovarian teratoma [1]. Since then, approximately 600 cases of this clinicopathologic entity have been reported [2-5]. The clinical presentation is related to psychiatric complaints and movement disorders [6]. Although this disease is commonly encountered in young women with ovarian teratoma, reports involving children and men with no tumors are also available [7-10]. Ictal arrhythmias, most likely originating from the sinus node, are well-established events in patients with autoimmune encephalitis. Prior anecdotal clinical evidence also suggests an increased propensity of developing cardiac arrhythmia secondary to anti-NMDA receptor encephalitis [11]. However, torsades de pointes with cardiac arrest is an extremely rare occurrence in these patients.

We present here a novel case of anti-NMDA receptor encephalitis where the patient experienced an episode of ictal torsades de pointes with cardiac arrest. Cardiac pathology was successfully managed as a part of the central nervous system (CNS) pathology. Aggressive immunomodulation and surgical removal of underlying ovarian teratoma resulted in a cure for both encephalitis and cardiac instability. Therefore, we speculate that prompt treatment of encephalitis may resolve the dysrhythmias without the need for permanent pacing. Additionally, we review the pertinent medical literature for cardiac complications in patients with anti-NMDA receptor encephalitis.

## Case presentation

A 34-year-old female with a history of mild depression was referred to our hospital from a psychiatric facility for medical evaluation of acute-onset bizarre behavior and intermittent unresponsiveness. The patient was occasionally redirectable and answered questions but she often appeared withdrawn, hallucinating (auditory), internally stimulated, and exhibited echolalia. Per collateral information, she was under extreme stress at work. She had experienced two similar self-limiting episodes in the past year. Her home medications included escitalopram, and she did not use dietary supplements. She denied fever, nausea, vomiting, and diarrhea. Her family history was negative for psychiatric disorders. Upon initial evaluation, she was completely withdrawn, tachycardic (130 beats/minute), hypertensive (174/115 mmHg), and afebrile. She had occasional non-rhythmic face twitching, hyperreflexia, and down-going toes.

**Figure 1:**
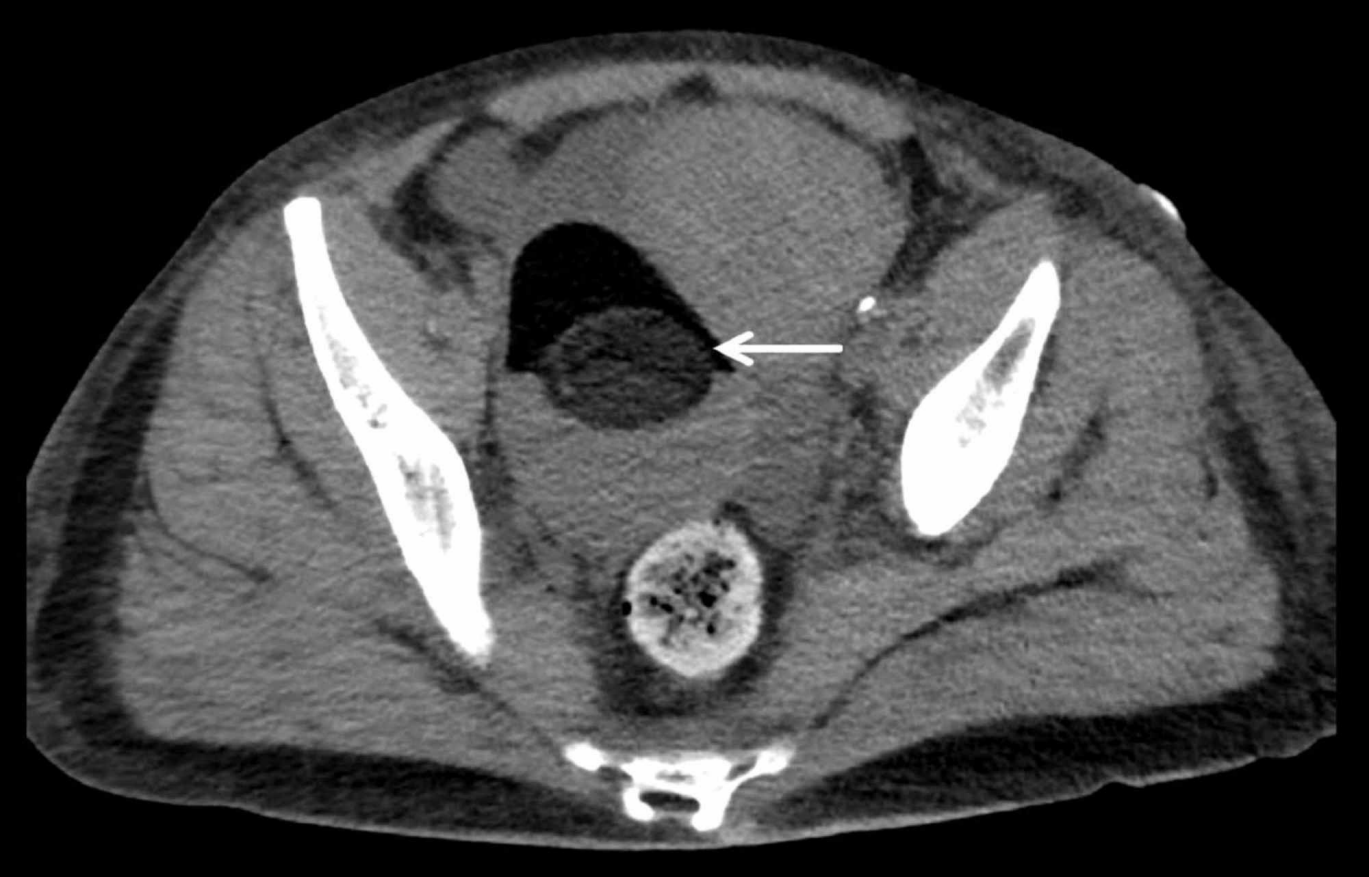
Computed tomography scan of the abdomen and pelvis showing a 9.2 x 6.5 x 7.8-cm mass (arrow) in the right adnexa, likely arising from the right ovary containing fat, fluid, and calcific densities (axial view).

Laboratory studies were significant for hypokalaemia (3.1 mmol/L), increased anion gap (18 mEq/L), and hyperglycemia (blood sugar fasting: 123 mg/dL). Her liver function testing showed alanine aminotransferase (ALT) 194 IU/L, aspartate aminotransferase (AST) 327 IU/L, total bilirubin 2.2 mg/dL, and direct bilirubin 0.9 mg/dL. The thyroid-stimulating hormone level was 2.58 mIU/mL. Her serum magnesium level was 0.9 mmol/L. Blood analysis for alcohol and acetaminophen was negative. The urine pregnancy test was negative. Urine drug screening and analysis detected benzodiazepines and ketones, respectively. A computed tomography (CT) scan of the head was negative for gross acute intracranial hemorrhage, mass effect, or hydrocephalus. CT abdomen and pelvis without contrast revealed a 9 x 8-cm complex mass in the right adnexa, which mimicked a teratoma (Figure 1).

**Figure 2:**
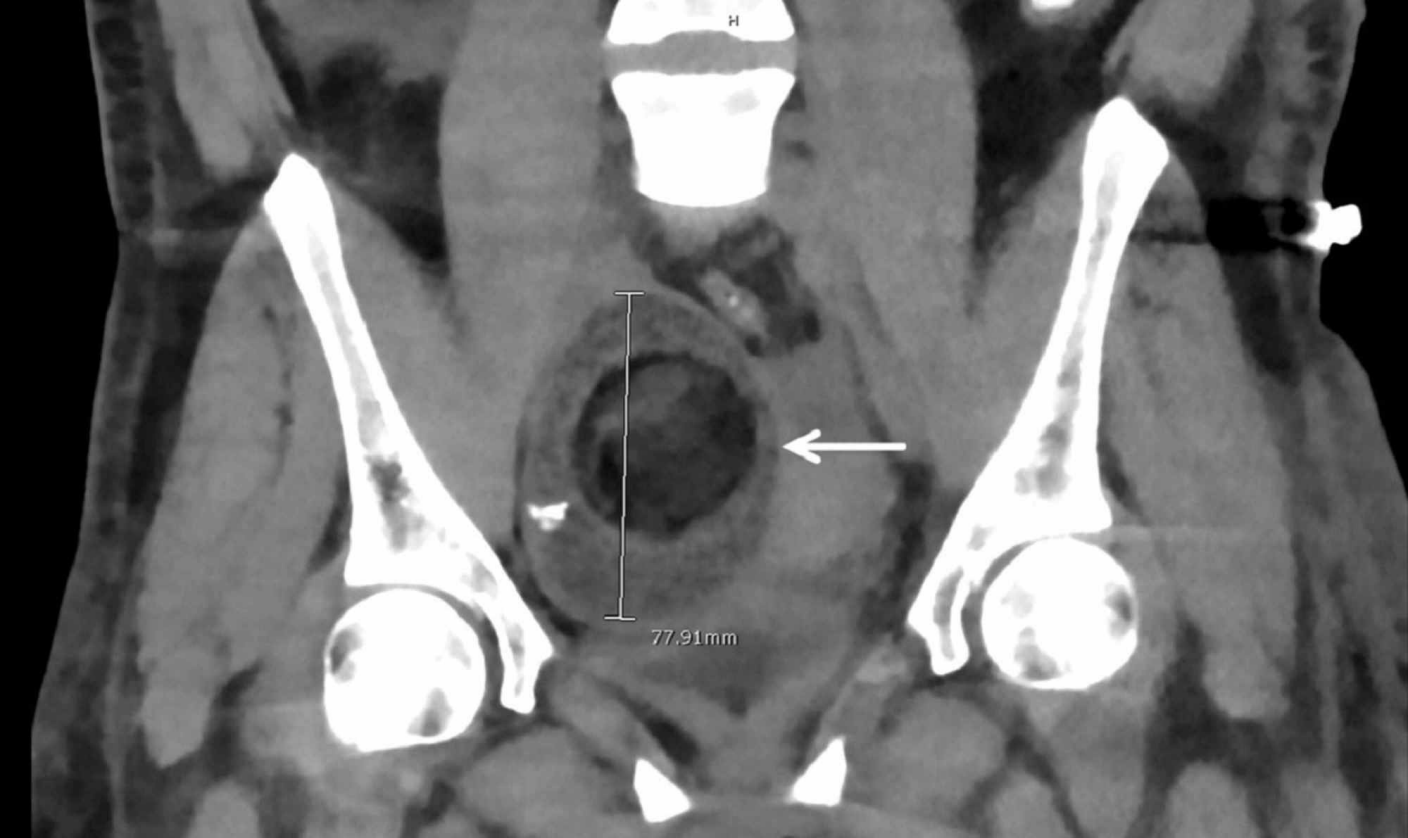
Computed tomography scan of the abdomen and pelvis showing the teratoma (arrow; coronal view).

The coronal view of the CT scan of the abdomen and pelvis showed the longest diameter of the teratoma to be 77.91 mm (Figure 2).

**Figure 3:**
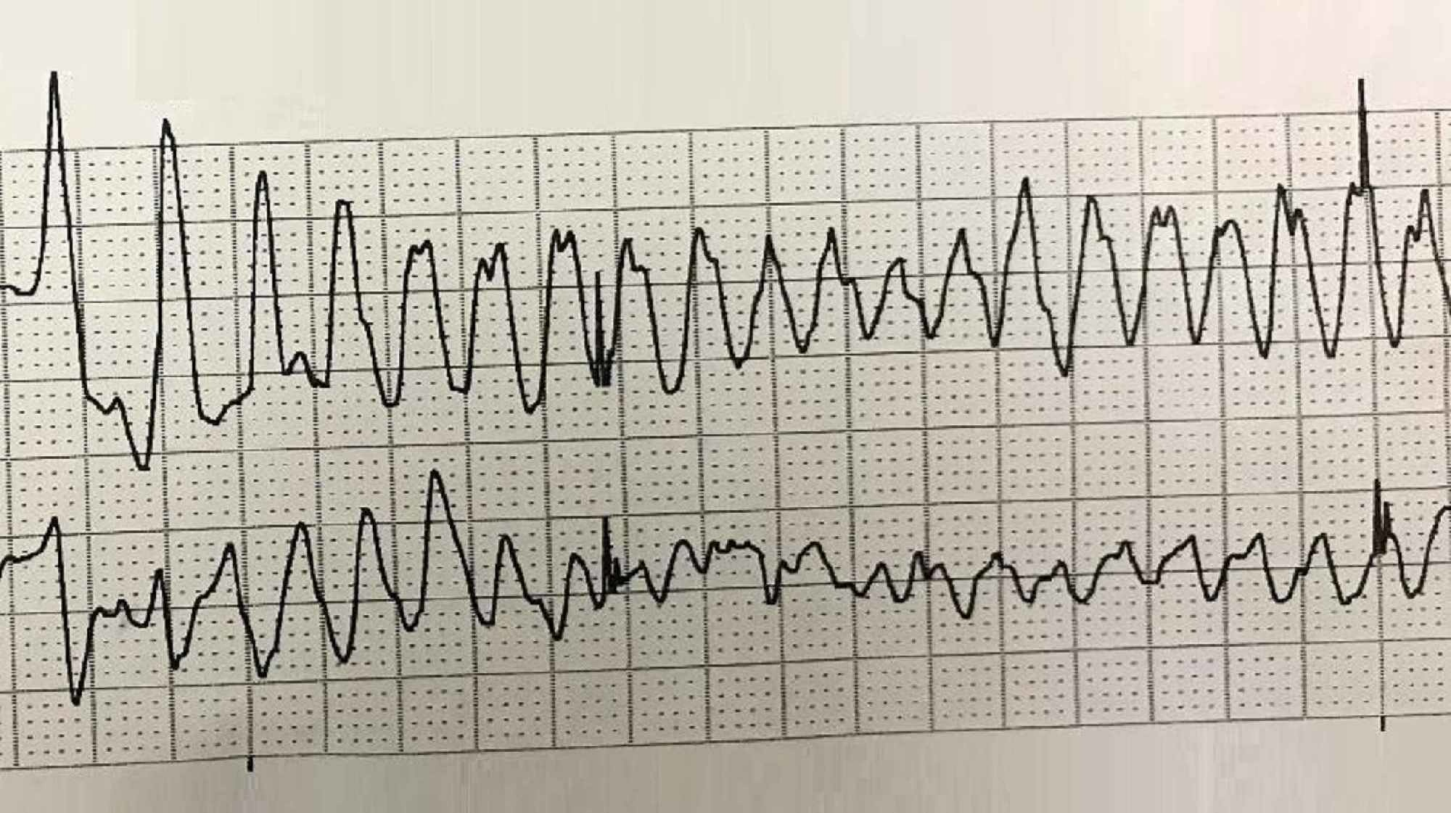
Electrocardiogram showing polymorphic ventricular tachycardia (torsades de pointes) during the cardiac arrest.

Findings of volume overload with ascites, mesenteric edema, and anasarca were also noted. During the CT abdomen study, the patient unusually developed a tonic-clonic seizure with subsequent cardiac arrest. Her initial cardiac rhythm showed polymorphic ventricular tachycardia, consistent with torsades de pointes (Figure 3).

Intravenous magnesium and epinephrine were administered. Spontaneous circulation successfully returned after two defibrillation attempts. Electrocardiogram then showed sinus tachycardia (120 beats/minute) and prolonged QTc (607 milliseconds). She was placed on targeted-temperature monitoring and was transferred to the coronary care unit (CCU).

During her CCU stay, she was started on lacosamide. On further workup, serum human immunodeficiency virus, rapid plasma reagin (RPR) titers, QuantiFERON®-TB Gold blood test (QIAGEN, Hilden, Germany) acute hepatitis panel, antinuclear antibody (ANA) test, rheumatoid factor, anti-glutamic acid decarboxylase (anti-GAD) antibodies, and anti-thyroid peroxidase (anti-TPO) antibodies were negative. Furthermore, vitamin B12 and folate levels were within normal limits. Transthoracic echocardiography revealed normal left ventricular (LV) size and wall thickness, with moderately reduced LV systolic function (ejection fraction = 35%-40%), which was potentially attributable to brief hypoperfusion myocardial injury during cardiac arrest. Right ventricle (RV) had normal size and function, with no significant valve abnormality. Cardiac catheterization showed angiographically normal coronary arteries. A long-term monitoring electroencephalogram (EEG) demonstrated diffuse slowing but no epileptiform discharges. Thereafter, an uneventful lumbar puncture was performed. Cerebrospinal fluid (CSF) samples for anti-NMDA receptor antibody testing, polymerase chain reaction (PCR) testing for viral etiologies, immunoglobulin G (IgG) index, oligoclonal bands, and cultures came out inconclusive. Serum ceruloplasmin, 24-hour urine copper, and ophthalmology evaluation ruled out Wilson’s disease. Magnetic resonance imaging (MRI) brain without contrast revealed several scattered, tiny, embolic-appearing infarcts.

Based on the presence of clinical features consistent with the probable criteria, CT findings showing an adnexal mass, most likely a teratoma, and after the exclusion of other possible etiologies, a diagnosis of anti-NMDA receptor encephalitis was established. She underwent one plasmapheresis session concurrently with intravenous immunoglobulin (IVIG) therapy. Subsequently, a spontaneous awakening trial was attempted but she became agitated and extubated herself. She continued to demonstrate QTc prolongation (519 milliseconds) and exhibited intermittent hallucinations, agitation, and confusion. Empiric antibiotic therapy was discontinued due to negative culture results (cerebrospinal fluid, blood, urine, and sputum). PCR testing for the varicella-zoster virus, cytomegalovirus, Epstein-Barr virus, and human herpesviruses was negative. She completed her IVIG treatment without any complications. Subsequently, laparoscopic tumor excision with right salpingo-oophorectomy was performed. Pathologic examination of the resected specimen confirmed the diagnosis of a teratoma. She had an uneventful post-operative recovery and was discharged from the hospital in a stable condition. On her six-week follow-up, her symptoms of psychosis and hallucinations were completely resolved. She resumed daily activities and her job.

## Discussion

Anti-NMDA receptor encephalitis is an acute-onset autoimmune neuropsychiatric disorder [1-2]. It has been described as the most common cause of antibody-associated encephalitis [3-4]. In one study conducted in the United Kingdom, anti-NMDA receptor antibodies were found in 1% of the patients with encephalitis admitted to intensive care units [5]. It was initially described as a paraneoplastic syndrome in young females with underlying teratomas. However, newer data demonstrate that only 38% of patients with anti-NMDA encephalitis have underlying neoplasms, most commonly ovarian teratomas. Other relatively rare malignancies include extra-ovarian teratomas, testicular germ-cell tumors, small-cell lung cancer, and Hodgkin’s lymphoma [2,6]. Anti-NMDA receptor encephalitis has a well-established clinical course. Over 80% of patients develop a prodromal viral-like illness lasting up to a week. This phase is followed by a myriad of psychiatric symptoms such as anxiety, agitation, bizarre behavior, vivid hallucinations, and delusions. Most patients also manifest a spectrum of neurological symptoms like dyskinesias, seizure, stupor, and catatonia. Approximately 60% of patients demonstrate features of autonomic instability in the form of heart rate, blood pressure, and body temperature variations along with urinary incontinence [7].

NMDA receptors belong to a class of glutamate receptors, one of the main excitatory neurotransmitters in the brain that play a role in vital brain functions related to memory and cognition. These receptors are made up of subunits known as NMDA receptor 1 (NR1), NR2 (A, B, C, and D), and NR3 (A and B). Anti-NMDA receptor encephalitis is an autoimmune syndrome and this statement is supported by several observations: the major antigen for antibody response seen in this disease is NR1/NR2B, which is predominantly expressed in the forebrain and hippocampus [1]. Antibody titers against the NR1/2 subunits of the NMDA receptors correlate with the disease severity and progression. These antibodies are usually detected in the affected patient’s serum but are frequently found at a higher concentration in cerebrospinal fluid (CSF), which implicates their intrathecal synthesis. The exact underlying cellular processes leading to the synthesis of these antibodies remain poorly understood. However, the presence of these antibodies recognizes the external domain of NR1 receptors and causes a decrease in the NMDA receptor cluster density. These findings strongly predict the antibody-mediated pathogenesis of anti-NMDA receptor encephalitis [8-9].

Occasionally, an initial routine CSF analysis can be normal or it may reveal lymphocytic pleocytosis and oligoclonal bands in these patients. Magnetic resonance imaging (MRI) is unremarkable in half of the patients with anti-NMDA receptor encephalitis. EEG may show nonspecific, slow, disorganized wave patterns. Detection of IgG antibodies to the NR1 subunit of the NMDA receptor in serum or CSF is highly specific for anti-NMDA receptor encephalitis, but its sensitivity remains questionable. In one study, 15% of patients fulfilling the clinical criteria for anti-NDMA receptor encephalitis were negative for anti-NMDA receptor antibodies. This observation supports the findings of our patient, who was diagnosed as anti-NMDA receptor encephalitis based on the probable clinical criteria but anti-NDMA receptor antibodies were not detected in serum or CSF [10].

Autonomic instability and cardiac dysrhythmias are established features of anti-NMDA receptor encephalitis. Approximately 66% of patients suffer from autonomic instability and about 33% develop cardiac dysrhythmias during the course of the disease. Up to 90% of rhythm disturbances originate from sinus node abnormalities. In our literature search, only one patient was identified, who experienced a short run of non-sustained ventricular tachycardia [11]. However, to the best of our knowledge, there are no previously reported cases of anti-NMDA receptor encephalitis developing ictal torsades de pointes leading to a cardiac arrest. Torsades de pointes or polymorphic ventricular tachycardia is usually caused by electrolyte abnormalities and drugs leading to prolonged QTc interval. It was notable that our patient had a serum potassium level of 3.1 mmol/L at presentation. Hypokalemia is a potential etiology of QTc prolongation and torsades de pointes. However, the serum potassium level has to be significantly low (less than 3 mmol/L) in order to cause clinically significant ventricular arrhythmias [12]. This patient was taking escitalopram as a prescription medication for her psychiatric symptoms. It is considered one of the possible causes of QTc prolongation but even in cases of escitalopram overdose, there has not been a single reported case of torsades de pointes in the published medical literature [13].

The exact pathophysiology of ictal cardiac dysrhythmias remains to be determined. It can potentially be a direct effect of seizure discharge on areas of the brain regulating sympathetic and parasympathetic supply to the heart. The insular, orbitofrontal, and anterior cingulate cortices and the amygdala are involved in the modulation of cardiac autonomic control and can be affected by the ictal discharge of cortical neurons. However, panencephalitis caused by the anti-NMDA receptor antibodies may also contribute to the central autonomic instability that can lead to tachyarrhythmias, bradyarrhythmias, and asystole [14].

Patients with anti-NMDA receptor encephalitis are usually treated with immunotherapy and potential tumor resection. Tumor resection usually results in the resolution of this autoimmune phenomenon. However, these patients commonly require admission in the intensive care unit for recurrent seizures and autonomic instability leading to cardiac dysrhythmias, rendering them unfit for early tumor removal. In these patients, the management of ictal arrhythmias is not extensively studied. In the presence of reversible brain pathologies like autoimmune encephalitis, the treatment of seizures and encephalitis may be sufficient to control cardiac rhythm problems [15]. This patient was initially started on aggressive immunomodulatory treatment with plasmapheresis and IVIGs. Although cardiac arrest with torsades de pointes rhythm was treated according to standard guidelines, she continued to have QTc interval prolongation. Subsequent surgical removal of the ovarian teratoma resulted in a cure of not only neuropsychiatric symptoms but also of the associated QTc prolongation and cardiac instability. The specific management of such patients remains controversial and further studies are warranted to outline efficient management algorithms.

## Conclusions

Anti-NMDA receptor encephalitis should be suspected in patients with acute-onset neuropsychiatric illnesses, especially in females with underlying gynecological malignancies. Seizures and ictal arrhythmias requiring cardiopulmonary resuscitation are possible sequelae of anti-NMDA receptor encephalitis. The present paper demonstrates that torsades de pointes can be an unusual arrhythmic complication in patients with anti-NMDA receptor encephalitis and treatment of underlying neurological pathology may cure serious cardiovascular instability.
